# Editorial: Neuroendocrine-Immunological Interactions in Health and Disease

**DOI:** 10.3389/fendo.2021.718893

**Published:** 2021-09-06

**Authors:** Ana Rosa Pérez, Clarissa M. Maya-Monteiro, Vinicius Frias Carvalho

**Affiliations:** ^1^Instituto de Inmunología Clínica y Experimental de Rosario, Consejo Nacional de Investigaciones Científicas y Técnicas (CONICET), Rosario, Argentina; ^2^Facultad de Ciencias Médicas, Universidad Nacional de Rosario, Rosario, Argentina; ^3^Laboratory of Immunopharmacology, Oswaldo Cruz Institute (IOC), Oswaldo Cruz Foundation (FIOCRUZ), Rio de Janeiro, Brazil; ^4^Department of Endocrinology and Metabolism, Amsterdam University Medical Centers (Amsterdam UMC), Amsterdam, Netherlands; ^5^Metabolism and Reward Group, Netherlands Institute for Neuroscience (NIN), Amsterdam, Netherlands; ^6^Laboratory of Inflammation, Oswaldo Cruz Institute (IOC), Oswaldo Cruz Foundation (FIOCRUZ), Rio de Janeiro, Brazil

**Keywords:** neuroendocrine, immunoendocrine, neuroimmune, metabolism, hormones, adipocytokines, cytokines

Historically, scientists have delimited the immune, endocrine, and neural systems to study physiology and disease (**Figure 1**). Although questions relating to whether there is a strict boundary between these systems do persist today, we do not believe in that. Biology does not seem to “respect” the established limits between these systems. Indeed, since Claude Bernard’s early studies in physiology, we know that the different organs and systems must communicate in an integrative way to maintain homeostasis. During recent decades, we have investigated these systems in an integrative way, since both the tools and the information to perform these studies are now available. In this Research Topic, we gathered diverse studies that increase our knowledge about the complex interactions among the immune, endocrine, and neural systems in both homeostasis and disease, and the potential therapeutic or disrupting agents of these circuits.

**Figure 1:**
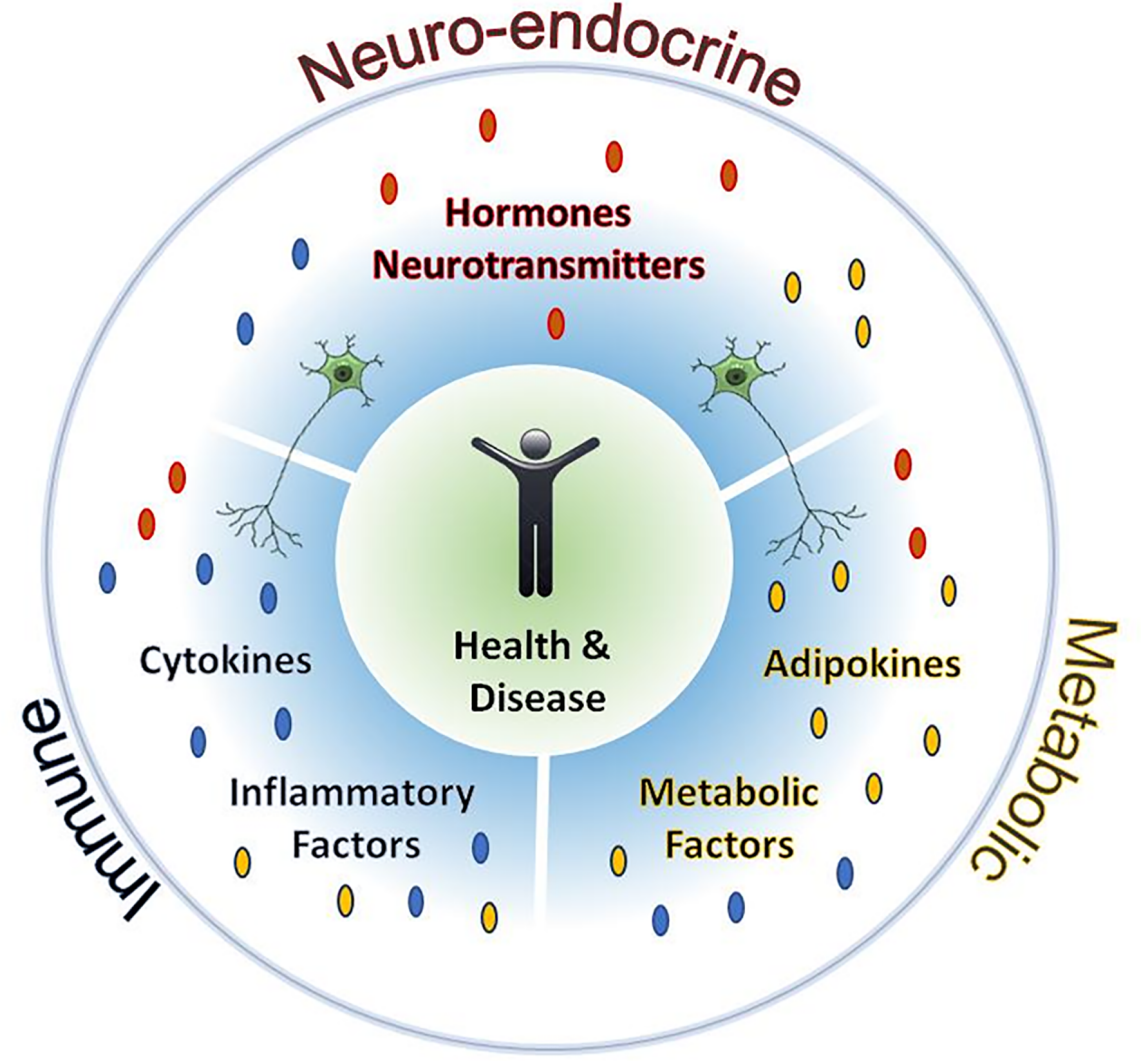
Neuroendocrine, immune and even metabolic systems are interconnected by a profuse network of mediators.

The similarities between nervous, immune, and endocrine systems are remarkable, and there are a number of shared mechanisms, agents, and receptors. As an example, cytokines and hormones are both important mediators of the hypothalamus–pituitary–adrenal (HPA) and hypothalamus–pituitary–thyroid (HPT) axes in response to stress and promoting immune modulation. Activation of the HPA axis triggers the synthesis of hypothalamic corticotropin-releasing hormone (CRH), followed by the release of the pituitary adrenocorticotropic hormone (ACTH) and activation of adrenal glands to secrete cortisol and dehydroepiandrosterone (DHEA). An imbalance of these components affects both positive and negative regulatory loops, leading to the predisposition for, and/or exacerbation of several infectious diseases. This imbalance was well described by Fernandez et al. as they studied the immune-endocrine system in the presence of tuberculosis and diabetes comorbidity, and demonstrated opposite effects on DHEA and cortisol. In humans, the immune response developed against *B. abortus* is also influenced by DHEA and cortisol secretions. Here, Gentilini et al. evaluated the consequence of both adrenal steroids on synoviocytes during *B. abortus* infection, contributing to knowledge about this infectious osteoarthritis. Furthermore, infectious-driven immune stimulation could sustain glucocorticoid production through changes in the intra-adrenal catabolic pathways or induced cellular damage, as shown by Silva Barbosa et al. and by Chen et al. in experimental models of Chagas disease and sepsis, respectively.

It is known that endogenous hypercortisolism or exposure to exogenous glucocorticoids can lead to Cushing’s syndrome. Liu et al. report on a rare case of an ectopic ACTH-dependent Cushing’s syndrome and proposed a mechanism for this unique clinical phenotype. Despite the adverse effects that sustained hypercortisolism may cause, the chronic use of corticosteroids to treat inflammatory diseases is widely accepted in clinical settings. As reported here, Ferreira et al. revealed a promising use of flunisolide as a pharmacological treatment for silicosis. Another important neuro-endocrine circuit, essential for dealing with stress, is the sympatho-adrenal system. Pilipovic et al. summarized the data, pointing to an immunopathogenic role for the sympathoadrenal axis in the pathogenesis of experimental autoimmune encephalomyelitis and multiple sclerosis.

Activation of the HPT axis induces the production of hypothalamic thyrotropin-releasing hormone (TRH), followed by the release of the thyroid-stimulating hormone (TSH), and synthesis of thyroid hormones. Hashimoto’s encephalopathy (HE) is an unusual neuropsychiatric syndrome characterized by elevated levels of autoantibodies against several thyroid antigens. Yu et al. described a case report of HE in a patient whose clinical symptoms and laboratory test results mimicked viral encephalitis. The effective diagnosis enabled the successful use of immunosuppressive therapy. In addition, Ferraris et al. showed that octyl methoxycinnamate, one of the most used UV filters, disrupted thyroid regulation and modulated the immune system in mice pups.

The adipose tissue is considered to be an immunoendocrine organ capable of producing a wide variety of mediators; the main adipose tissue specific cytokines, the adipocytokines, are leptin and adiponectin. Disruption of adipose tissue homeostasis can lead to alterations in many critical aspects of immunity. Pesce Viglietti et al. showed how *B. abortus* can modulate the transcription of adipocytokines and affect the process of adipogenesis both directly and indirectly. Palhinha et al. described for the first time that leptin autocrine signaling pathways induce adipogenesis and proinflammatory profile. This effect may have particular importance during obesity when leptin central nervous system signaling is defective. Amorim et al. showed that leptin can also trigger an eosinophilic inflammatory response *in vivo*, by an indirect mechanism dependent on the activation of resident mast cell secretory activity and mediated by TNFα, CCL5, and PGD2.

Immunometabolism can be regarded as a branch of neuroendocrine-immunology examining the crosstalk between metabolism and the immune response. Smitka et al. summarized evidence showing that the gut microbiome is a potential modulator of adipose tissue, energy homeostasis, and appetite satiety in eating disorders, like anorexia and bulimia. The work from Silva et al. proposed that short-chain fatty acids from gut microbiota can be used as a therapy for central nervous system (CNS) disorders through its capacity to regulate the neuro-immunoendocrine function. Milanova et al. showed that hypothalamic microglia from high-fat diet fed mice were activated and lost rhythm, displaying immuno-metabolic functions different from those observed in microglia from control animals. Huang et al. demonstrated that high glucose or glucose fluctuations caused M2 phenotype polarization in a microglial cell line *in vitro*. They also showed that miR-146a overexpression inhibited high-glucose-induced M1/M2 polarization transitions in those cells.

In literature, activation of immune system secondary lymphoid organs (SLOs) has been observed to coincide with the decrease in noradrenergic activity and/or retraction of sympathetic fibers. Bottasso *(part I) and* Bottasso *(part II)* introduced a challenging hypothesis for the existence of a neural plasticity program in the sympathetic fibers innervating both SLOs and non-lymphoid peripheral tissues during inflammation. According to the author, this plasticity implies a retraction and degeneration of sympathetic fibers during immune activation and their re-generation once homeostasis is re-established. The activation of the immune system can be important in the disruption of neural circuits and induction of nervous system disorders. In this regard, Li et al. showed that IFN-α, administered by i.c.v. routes induced depression-like behavior in monkeys. This was associated with a dysfunction in some monoamine neurotransmitters founded in the cerebrospinal fluid. Stary et al. found that nursing in the post-partum period protected the mouse nervous system in a model of middle cerebral artery occlusion-induced stroke. They showed a reduced neurological deficit, lower pro-inflammatory cytokine levels, and lower migration of blood leukocytes into the brain, apparently by an increase of local oxytocin levels in the nursing mice. Along these lines, Kang et al. summarized the protective effects of exosomes on ischemic and hypoxic brain injury by inhibiting neuronal apoptosis, mediating axon reconstruction and neurogenesis, and alleviating inflammatory response and immune suppression.

Neuroendocrine disorders interfere with the interactions between the nervous and the endocrine systems, causing excessive or deficient hormone production with a negative impact on metabolism. The Prader-Willi syndrome (PWS) was the first neuroendocrine disease to be related to genomic imprinting errors. Costa et al. reviewed and summarized the disrupted genes related to the clinical phenotypes of PWS. Another important neuroendocrine disorder is Alzheimer’s disease (AD), where deficiency of the tau protein in the CNS is an important feature in AD pathology. Gonçalves et al. showed that an AD mouse model knockout for tau protein presented anxiety-related behavior and memory impairment. They also verified that the introduction of human tau, in tau knockout mice, did not restore anxiety or metabolic alterations and triggered insulin resistance and further impairments in learning and memory features. Finally, Alvarez-Herrera et al. summarize the peripheral immunological, endocrine, and intestinal microbiome changes induced by atypical antipsychotics used for the treatment of schizophrenia and other psychiatric disorders.

In recent decades, our understanding of the intricate network between nervous, immune, and endocrine systems, and the development of diseases has remarkably increased. However, extensive challenges remain in providing a more comprehensive picture. The articles presented in this Research Topic show the complex circuitry affecting the neuroendocrine-immunological interactions in different diseases and indicate future directions for research in this area.

## Author Contributions

All authors contributed equally to the Topic (Topic Image, ARP). All authors contributed to the article and approved the submitted version.

## Funding

This study obtained funding from Capes (Brazil), PICT 2016-0312 (Argentina).

## Conflict of Interest

The authors declare that the research was conducted in the absence of any commercial or financial relationships that could be construed as a potential conflict of interest.

## Publisher’s Note

All claims expressed in this article are solely those of the authors and do not necessarily represent those of their affiliated organizations, or those of the publisher, the editors and the reviewers. Any product that may be evaluated in this article, or claim that may be made by its manufacturer, is not guaranteed or endorsed by the publisher.

